# Philadelphia College of Pharmacy

**Published:** 1858-05

**Authors:** 


Philadelphia College of Pharmacy.—During the session of 1857–58, the number of matriculates at this old and flourishing Institution was 90. At the commencement, held on the evening of 10th March, the degree of Graduate in Pharmacy was conferred on 31 gentlemen, and the valedictory address was delivered by that accomplished pharmaceutist and lecturer, Professor Wm. Proctor, Jr.

